# Proteomic identification of OsCYP2, a rice cyclophilin that confers salt tolerance in rice (*Oryza sativa *L.) seedlings when overexpressed

**DOI:** 10.1186/1471-2229-11-34

**Published:** 2011-02-16

**Authors:** Song-Lin Ruan, Hua-Sheng Ma, Shi-Heng Wang, Ya-Ping Fu, Ya Xin, Wen-Zhen Liu, Fang Wang, Jian-Xin Tong, Shu-Zhen Wang, Hui-Zhe Chen

**Affiliations:** 1 Plant Molecular Biology & Proteomics Lab, Institute of Biotechnology, Hangzhou Academy of Agricultural Sciences, Hangzhou 310024, PR China; 2 National Key Laboratory of Rice Biology, China National Rice Research Institute, Hangzhou 310006, PR China

## Abstract

**Background:**

High Salinity is a major environmental stress influencing growth and development of rice. Comparative proteomic analysis of hybrid rice shoot proteins from Shanyou 10 seedlings, a salt-tolerant hybrid variety, and Liangyoupeijiu seedlings, a salt-sensitive hybrid variety, was performed to identify new components involved in salt-stress signaling.

**Results:**

Phenotypic analysis of one protein that was upregulated during salt-induced stress, cyclophilin 2 (OsCYP2), indicated that *OsCYP2 *transgenic rice seedlings had better tolerance to salt stress than did wild-type seedlings. Interestingly, wild-type seedlings exhibited a marked reduction in maximal photochemical efficiency under salt stress, whereas no such change was observed for *OsCYP2*-transgenic seedlings. *OsCYP2*-transgenic seedlings had lower levels of lipid peroxidation products and higher activities of antioxidant enzymes than wild-type seedlings. Spatiotemporal expression analysis of *OsCYP2 *showed that it could be induced by salt stress in both Shanyou 10 and Liangyoupeijiu seedlings, but Shanyou 10 seedlings showed higher *OsCYP2 *expression levels. Moreover, circadian rhythm expression of *OsCYP2 *in Shanyou 10 seedlings occurred earlier than in Liangyoupeijiu seedlings. Treatment with PEG, heat, or ABA induced *OsCYP2 *expression in Shanyou 10 seedlings but inhibited its expression in Liangyoupeijiu seedlings. Cold stress inhibited *OsCYP2 *expression in Shanyou 10 and Liangyoupeijiu seedlings. In addition, OsCYP2 was strongly expressed in shoots but rarely in roots in two rice hybrid varieties.

**Conclusions:**

Together, these data suggest that OsCYP2 may act as a key regulator that controls ROS level by modulating activities of antioxidant enzymes at translation level. OsCYP2 expression is not only induced by salt stress, but also regulated by circadian rhythm. Moreover, OsCYP2 is also likely to act as a key component that is involved in signal pathways of other types of stresses-PEG, heat, cold, or ABA.

## Background

Rice is a salt-sensitive cereal crop. High salinity may cause delayed seed germination, slow seedling growth, and reduced rate of seed set, leading to decreased rice yield. These disorders are generally due to the combined effects of ion imbalance, hyperosmotic stress, and oxidative damage. In the early period, rice can rapidly perceive a salt stress signal via plasma membrane receptors in root cells and can rapidly initiate an intracellular signal that modulates gene expression to elicit an adaptive response.

Functional genomics is an effective tool for identifying new genes, determining gene expression patterns in response to salt stress, and understanding their functions in stress adaptation. Initially, gene expression is examined at the mRNA level using large-scale screening techniques such as cDNA microarrays, serial analysis of gene expression, and cDNA-amplified fragment-length polymorphism. cDNA microarrays containing 1728 cDNAs were used to analyze gene expression profiles during the initial phase of salt stress in rice roots, and found that approximately 10% of the transcripts in Pokkali were significantly upregulated or downregulated within 1 h of salt stress [1]. To date, cDNA microarray analyses have identified approximately 450 salt-responsive unigenes in shoots of the highly salt-tolerant rice variety, Nona Bokra, and most of them were not known to be involved in salt stress [2]. In addition, forward and reverse genetics have identified gene functions during salt stress. Interestingly, map-based cloning was used to isolate a rice quantitative trait loci gene, *SKC1 *that encoded an HKT-type transporter selective for Na^+^. Analysis of transgenic rice plants with loss-of-function or gain-of-function phenotypes that were changed by forward and reverse genetics revealed that *SKC1 *was involved in regulating K^+^/Na^+ ^homeostasis under salt stress [3]. Also, in *Arabidopsis*, overexpression of *SOS1*, which encoded a plasma membrane Na^+^/H^+ ^antiporter, improved salt tolerance [4].

Recently, proteome profiles of rice in response to salt stress were presented for various tissues or organs such as roots, leaf lamina, leaf sheaths and young panicles [5-8]. Although some differential proteins of interest have been identified, little is known about the functions of these proteins.

Here, OsCYP2, a salt-induced rice cyclophilin, was separated and identified by 2-DE, MALDI-TOF MS and ESI-MS/MS. OsCYP2 had peptidyl-prolyl cis-trans isomerase (PPIase or rotamase) activity that was specifically inhibited by cyclosporine A [9]. Moreover, *OsCYP2 *lacks introns, and the 5' end of transcript contains an AT-rich region, suggesting that *OsCYP2 *was likely to be preferentially translated during stress conditions [10]. Actually, *OsCYP2 *could respond to multiple environmental stresses such as high salt, drought, heat and oxidative stress. For example, heterologous expression of *OsCYP2 *was able to enhance ability of *E. coli *to survive, to complement the yeast mutant lacking native *OsCYP2 *and to improve the growth of wild type yeast under the above mentioned abiotic stresses [9]. In addition, significantly differential changes in transcript abundance of *OsCYP2 *were found in shoots of salt sensitive (IR64) and tolerant (Pokkali) rice cultivars at different developmental stages under normal and salt stress conditions [9].

We have therefore focused on the effect of *OsCYP2 *expression on salt tolerance in rice seedlings. Overexpression of *OsCYP2 *conferred salt tolerance in transgenic rice seedlings. Although *OsCYP2*-transgenic seedlings did not predominate over wild-type seedlings in ion homeostasis (K^+^/Na^+ ^ratio) and osmotic regulation (free proline), they displayed lower levels of lipid peroxidation products and higher activities of antioxidant enzymes than wild-type seedlings, suggesting that the involvement of *OsCYP2 *in the response of rice seedling to salt stress is required, but also it can enhance salt tolerance in transgenic rice seedlings by controlling ROS levels. In addition to salt stress, OsCYP2 can respond to other types of stresses, such as drought, heat and cold, indicating that OsCYP2 is likely to act as a general integrator of environmental stresses.

## Results

### Evaluation of the salt tolerance of two rice hybrid varieties

**Figure 1:**
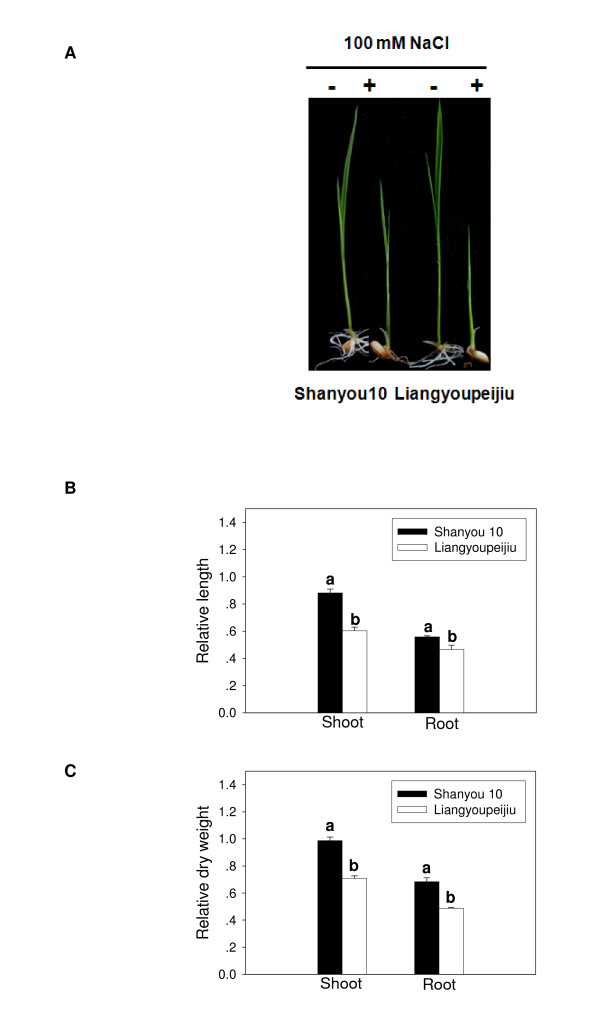
**Phenotypes of Shanyou 10 and Liangyoupeijiu seedlings after salt stress**. **(A) **Phenotypes of 10-day-old seedlings of Shanyou 10 and Liangyoupeijiu after salt stress (100 mM NaCl), as indicated by (+), or under normal conditions (no NaCl), as indicated by (-). **(B) **Relative length of shoots and roots of Shanyou 10 and Liangyoupeijiu seedlings. **(C) **Relative dry weight of shoots and roots of Shanyou 10 and Liangyoupeijiu seedlings. The distance from the basal part of shoot to tip of the longest leaf was calculated as the length of seedling. The percentage of relative FW, DW, or shoot/root length of the salt treated samples was calculated in relation to non-treated. Data represent the average of four treatments (mean ± S.E.). Identical letters above a pair of bars indicate that the values are not significantly different at the p = 0.05 level according to Duncan's multiple range test.

To compare the salt tolerance of the two rice hybrid varieties, Shanyou 10 and Liangyoupeijiu, relative length and dry weight of shoots and roots were determined after exposure to salt stress, respectively. The roots and shoots of Shanyou 10 were longer and heavier than those of Liangyoupeijiu (Figure 1B, C). Phenotypic analysis showed that Shanyou 10 seedlings grew faster than Liangyoupeijiu seedlings under salt stress conditions (Figure 1A), suggesting that Shanyou 10 seedlings were relatively more tolerant to salt.

### Separation and identification of differentially expressed salt-responsive proteins of rice seedlings

**Figure 2:**
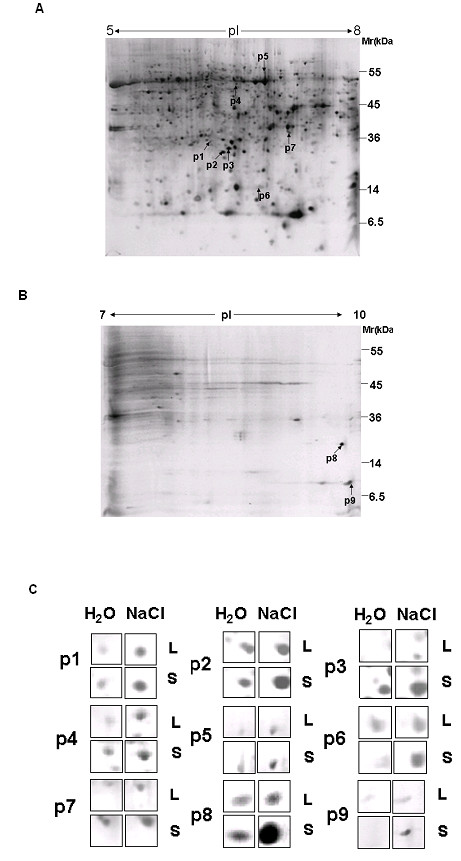
**Two-dimensional gel electrophoresis analyses of shoot proteins in Shanyou 10 and Liangyoupeijiu**. Rice shoot proteins separated by IEF/SDS-PAGE were stained with silver nitrate. Numbered spots represent proteins that were identified detailed in Table 1. **(A) **Total protein (120 μg) from rice shoots of Shanyou 10 treated with 100 mM NaCl was loaded onto a 17-cm IPG gel with pH 5-8. SDS-PAGE (12% gel) was used in the second-dimension separation. Gels were stained with silver nitrate solution. Numbers on the right represent apparent molecular masses. Numbers above gels represent isoelectric point range of separated proteins. **(B) **Total protein (200 μg) from rice shoots of Shanyou 10 treated with 100 mM NaCl was loaded onto a 17-cm IPG gel with pH 7-10. **(C) **The nine proteins of interest (p1-p9) that were differentially expressed are shown. S and L denote Shanyou 10 and Liangyoupeijiu, respectively.

To understand the differences between Shanyou 10 and Liangyoupeijiu at the protein expression level, 2-DE and MS were used to separate and identify differentially expressed salt-responsive proteins of rice seedlings in Shanyou 10 and Liangyoupeijiu. More than 1050 rice shoot proteins (more than 950 proteins from IPG5-8 and more than 100 proteins from IPG7-10) were detected by image match analysis. Of these, 34 proteins were up- or downregulated in response to salt stress. Nine upregulated proteins consistently showed significant and reproducible increases in abundance (1- to 4-fold) under NaCl stress (Figure 2A, B) and were selected for MALDI-TOF MS analysis. They were identified as a putative glutathione S-transferase, manganese superoxide dismutase, dehydroascorbate reductase (free radical scavenging), a putative phosphogluconate dehydrogenase (pentose phosphate pathway), putative l-aspartate oxidase (protein metabolism), putative cold shock protein-1(cold stress response), prohibitin (cell proliferation), a putative membrane protein (unknown function), a putative oxygen-evolving enhancer protein 3-1 (photosynthesis) and cyclophilin 2 (OsCYP2)(protein folding)(Table 1).

**Table 1:** Identification of shoot proteins of interest in hybrid rice by MALDI-TOF MS

Spot No.^a^	Apparent MW (KD)/pI^b^	MatchMW (KD)/pI^c^	MOWSE Score^d^	MOWSE Score for acceptance^e^	No. MP^f^	No. UMP^g^	Percent covered^h^	Accession No.^I^	Protein name
P1	26.4/5.80	25.64/5.82	61	60	5	16	26	Q9FUE6	Putative glutathione S-transferase
P2	23.8/5.85	24.98/6.50	69	60	6	36	43	AAA57131	manganese superoxide dismutase
P3	24.2/5.91	23.555/5.81	62	60	4	17	28	Q84UH5	dehydroascorbate reductase
P4	52.5/6.05	52.688/5.85	92	60	8	11	28	NP_910282	putative phosphogluconate dehydrogenase
P5	65.5/6.5	71.06/6.54	61	60	6	15	16	Q6Z836	Putative L-aspartate oxidase
P6	17.5/6.32	18.682/6.28	84	60	6	33	49	XP_479920	putative cold shock protein-1
P7	36.2/6.95	30.783/6.99	80	60	5	26	31	CAE76006	Prohibitin
P8	18.5/9.21	18.319/8.61	117	60	10	4	42	AAA57046	Cyclophilin 2 (OsCYP2)
P9	10.2/9.65	22.566/9.8	117	60	7	4	37	XP_478627	Putative oxygen -evolving enhancer protein 3-1

^a ^Spot Nos refer to spot number as given in Figure 2. ^b ^Apparent MW (KD)/pI: apparent molecular weight and pI values. ^c ^MatchMW (KD)/pI: match molecular weight and pI values. ^d ^MOWSE Score: Scores given in MASCOT database. ^e ^MOWSE Score for acceptance: protein scores greater than 60 are significant (p < 0.05). ^f ^No. MP: number of matched peptides. ^g ^No. UMP: number of unmatched peptides. ^h ^Percent covered: percent of all match peptide sequences to OsCYP2 sequence. ^I ^Accession No.: Accession number in NCBI database.

The p8 protein spot in Figure 2B was selected for further analysis using ESI-MS/MS to determine peptide sequence. Three peptides from the p8 spot were sequenced and matched to OsCYP2 in the MASCOT database (Table 2). Two peptides (m/z 1424.64 and 1656.64) were found in matched peptides from PMF (Additional file 1). These results identified the p8 spot as OsCYP2. The other protein spots were also validated using ESI-MS/MS (Additional file 2).

**Table 2:** Identification of peptides from OsCYP2 (p8 protein spot) by MALDI-TOF-MS and ESI-MS/MS

**Peptide no**.	Match peptide sequences	Methods of identification		PercentCovered (%)^c^	Modifications	Ion score	Ion score for acceptance
						
		MALDI-TOF MS	ESI-MS/MS				
1	VFFDMTVGGAPAGR	+^a^	+	8.14	None^d^	64	38
2	TAENFR	+	-^b^	3.49	None	ND^e^	ND
3	TAENFRALCTGEK	+	-	7.56	None	ND	ND
4	GSTFHR	+	-	3.49	None	ND	ND
5	VIPEFMCQGGDFTR	+	+	8.14	Carbamidomethyl (C)	79	38
6	GNGTGGESIYGEK	+	-	7.56	None	ND	ND
7	GNGTGGESIYGEKFADEVFK	+	-	11.63	None	ND	ND
8	FADEVFK	+	-	4.07	None	ND	ND
9	HVVFGR	+	-	3.49	None	ND	ND
10	GGSTA KPV VIADCGQ LS	-	+	9.88	Carbamidomethyl (C)	48	38

^a ^+: positive match. ^b ^-: no match. ^c ^percent covered: percent of peptide sequence to OsCYP2 sequence. ^d ^None: without modifications. ^e ^ND: not determined.

^f ^Ion score for acceptance: individual ion score > 38 indicate identity or extensive homology (p < 0.05).

**Figure 3:**
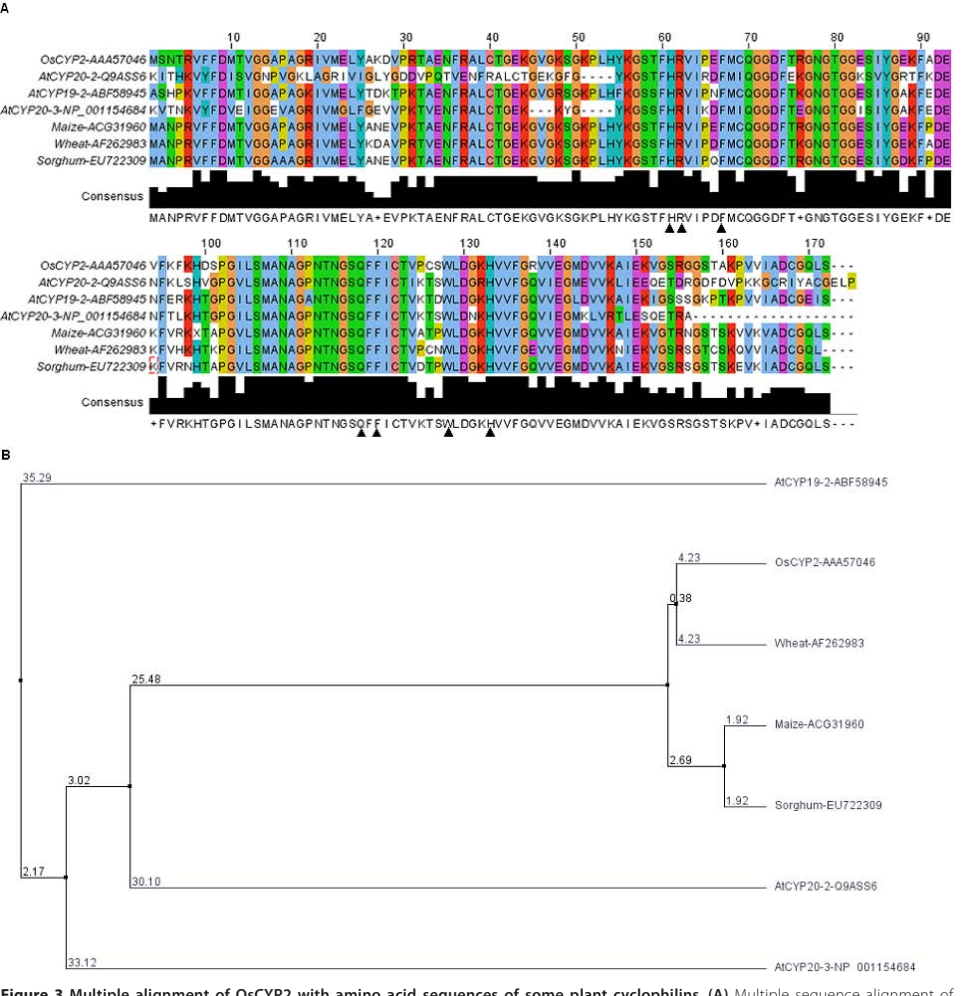
**Multiple alignment of OsCYP2 with amino acid sequences of some plant cyclophilins**. **(A) **Multiple sequence alignment of OsCYP2 with cyclophilins of various plant species by the Jalview multiple alignment editor. Seven residues (His-61, Arg-62, Phe-67, Gln-118, Phe-120, Trp-128 and His-33) associated with PPIase catalysis are marked by filled triangle (▲). Three of these, His-61, Arg-62 and Phe-120, are extremely important for PPIase activity of OsCYP2. The residue Trp-128 is a binding site of OsCYP2 with cyclosporin A (CsA). **(B) **Dendrogram showing phylogenetic distance among plant cyclophilins according to average distance using percentage identity.

*OsCYP2 *(accession no. AAA57046) was predicted to encode a protein of 172 amino acids with a molecular mass of 18.6 kDa and a *pI *of 8.61.In the conserved region of OsCYP2, the residues His-61, Arg-62, Phe-67, Gln-118, Phe-120, Trp-128 and His-33 appeared to be associated with PPIase catalysis. Three of these, including His-61, Arg-62 and Phe-120, are most essential for PPIase activity of OsCYP2. The residue Trp-128 is a binding site of OsCYP2 with immunosuppressant cyclosporin A (Figure 3A). OsCYP2 had significant homology with other known cyclophilins from various plant species (Figure 3A). The deduced amino acid sequence of OsCYP2 displayed higher identity with the cyclophilins of three cereal crops, *T. aestivum*, *Zea mays *and *Sorghum bicolor *(86% each), while OsCYP2 showed relatively lower identity with three cyclophilins of *Arabidopsis*, including AtCYP19-2 (78%), AtCYP20-2 (63%) and AtCYP20-3 (58%). Moreover, a closer relationship between OsCYP2 and the cyclophilins of three cereal crops was observed compared to *Arabidopsis *(Figure 3B).

### Phenotypic identification of OsCYP2 transgenic rice seedlings under salt stress

**Figure 4:**
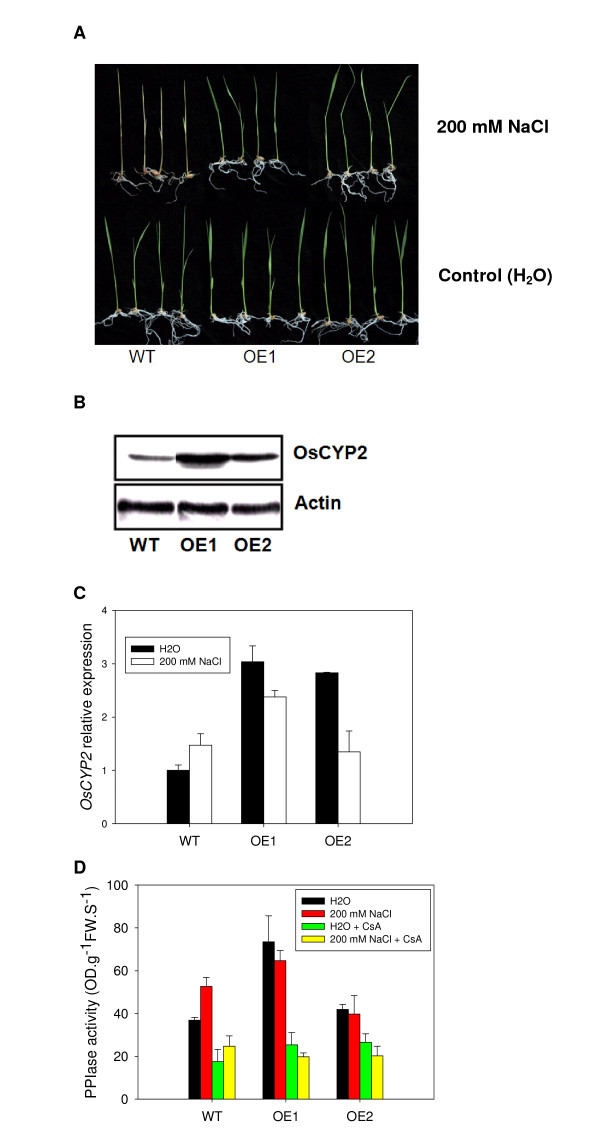
**Phenotypes of rice seedlings under salt stress**. **(A) ***OsCYP2 *transgenic rice lines showed salt tolerant phenotypes. Ten-day-old rice seedlings were treated with 200 mM NaCl. After 5 days, phenotypes of rice seedlings were observed. WT represents the wild-type seedling, *Aichi ashahi *that was used as a reference rice cultivar. **(B) **Western blot showed OsCYP2 overexpression in two *OsCYP2 *transgenic lines (OE1 and OE2). The housekeeping protein, Actin (Os03g0718100), was used as equal loading control. **(C) **Real time PCR exhibited differential expression pattern of *OsCYP2 *between WT and *OsCYP2 *transgenic lines (OE1 and OE2) under salt stress. Ten-day-old rice seedlings were treated for 1 d with 200 mM NaCl. An actin gene, *Os03g0718100*, was used as internal standard. **(D) **The altered activity of PPIase was found in WT and *OsCYP2 *transgenic lines (OE1 and OE2) under salt stress. Ten-day-old rice seedlings were treated for 1 d with 200 mM NaCl. Cyclosporin A (CsA) was able to partly inhibit the activity of PPIase.

To understand the response of transgenic rice seedlings with *OsCYP2 *overexpression to salt stress, we introduced this gene into wild-type rice (*O. sativa *cv. Aichi ashahi) to obtain T3 transgenic seedlings with single copy insertion (Additional file 3). Ten-day-old transgenic and wild-type seedlings were treated with 200 mM NaCl. After 5 days, leaves of wild-type seedlings exhibited the chlorotic phenotype, and in some cases died, whereas leaves of the transgenic seedlings remained green (Figure 4A). Similar phenotypes were observed in three-week-old wild type and transgenic seedlings treated with 150 mM NaCl for 7 d under water culture (Additional file 4). Significantly, two transgenic lines (OE1and OE2) showed *OsCYP2 *overexpression under normal condition compared to wild-type (Figure 4B, C). Although *OsCYP2 *expression was inhibited in two transgenic lines and was induced in wild type under salt stress, salt-stressed seedlings of two transgenic lines showed close or higher levels of *OsCYP2 *expression to or than that of wild type (Figure 4C). Similarly, two transgenic lines showed higher levels of PPIase activity under normal condition compared to wild type. Salt-stressed seedlings of wild type exhibited higher level of PPIase activity than unstressed seedlings, while no significant changes in levels of PPIase activity were found between salt-stressed and unstressed seedlings of two transgenic lines. Salt-stressed seedlings of two transgenic lines still kept close or higher levels of PPIase activity to or than that of wild type (Figure 4D). The addition of CsA significantly suppressed the PPIase activity of wild type and two transgenic lines (Figure 4D). Therefore, it was suggested that OsCYP2 was likely to play an important role in the response of rice seedlings to salt stress.

### Effect of salt stress on maximal photochemical efficiency (Fv/Fm) of OsCYP2 transgenic rice seedlings

**Figure 5:**
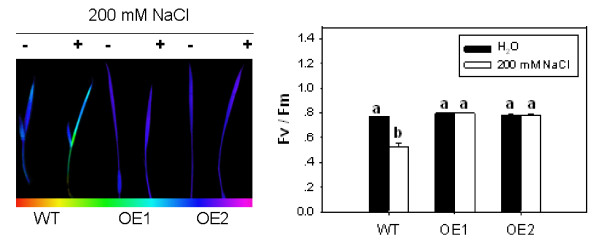
**Effect of salt stress on Fv/Fm of rice seedlings**. Under salt stress, lower Fv/Fm values were observed in wild-type seedlings, but no significant changes in Fv/Fm levels were observed in *OsCYP2 *transgenic rice lines. Ten-day-old rice seedlings of wild-type or *OsCYP2*-transgenic lines were used. Ten-day-old rice seedlings were treated with 200 mM NaCl for 24 h. Fluorescence from red to pink color represents values from minimal to maximal readout. Each value is the mean ± S.E. of six treatments. Identical letters above a pair of bars indicate there is no statistically significant difference among the transgenic lines at the p = 0.05 level according to Duncan's multiple range test.

Based on the observation that the *OsCYP2*-transgenic seedlings retained green color in their leaves, we speculated that *OsCYP2 *was likely to protect the photosynthetic components in rice leaves from oxidative stress caused by salt. We compared the effects of salt stress on the maximal photochemical efficiency (Fv/Fm) in *OsCYP2 *transgenic and wild-type seedlings. Salt stress significantly reduced the Fv/Fm in wild-type seedlings, but no significant change was observed in Fv/Fm for *OsCYP2 *transgenic seedlings (Figure 5), suggesting that *OsCYP2 *over-expression protected the photosynthetic components in rice leaves against oxidative stress.

### Effect of salt stress on lipid peroxidation and ROS scavenging in OsCYP2 transgenic rice seedlings

**Figure 6:**
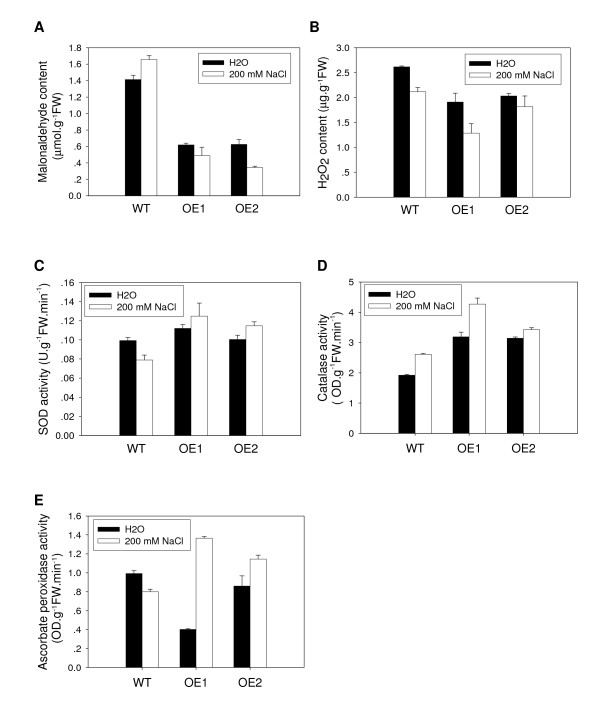
**Comparison of lipid peroxidation and ROS scavenging of *OsCYP2*-transgenic rice seedlings and wild-type seedlings under salt stress**. *OsCYP2*-transgenic rice seedlings had lower malonaldehyde (MDA) content and H_2_O_2 _and higher antioxidant enzyme activities than wild-type seedlings. Ten-day-old rice seedlings were treated with 200 mM NaCl for 24 h. The levels of MDA **(A) **and H_2_O_2 _**(B) **were determined with thiobarbituric acid (TBA) and ferric-xylenol orange complex, respectively. The activities of antioxidant enzymes SOD **(C)**, CAT **(D)**, and APX **(E) **were assayed. Each value was the mean ± S.E. of four treatments.

To further validate the protective effects of *OsCYP2 *on the photosynthesis machinery in rice leaves, we compared salt stress-induced changes in the lipid peroxidation product (MDA) and ROS scavenging in *OsCYP2*-transgenic and wild-type seedlings. The level of MDA in plant tissues was used as an indicator of lipid peroxidation [11]. Under normal conditions (no NaCl treatment), the MDA levels were lower in *OsCYP2*-transgenic seedlings than in wild-type seedlings (Figure 6A). By comparison, under salt stress (200 mM NaCl), the MDA levels were significantly reduced in *OsCYP2*-transgenic seedlings, whereas the MDA levels increased in wild-type seedlings (Figure 6A), indicating that *OsCYP2 *over-expression could decrease lipid peroxidation levels in transgenic rice seedlings.

Stress-induced H_2_O_2 _accumulation could increase lipid peroxidation [12]. Under normal conditions, *OsCYP2*-transgenic rice seedlings contained lower H_2_O_2 _levels than wild-type seedlings. At 24 h after treatment with 200 mM NaCl, each type of seedlings exhibited a decrease in H_2_O_2 _levels (Figure 6B). Similarly, the H_2_O_2 _levels in *OsCYP2*-transgenic rice seedlings were lower than that in wild-type seedlings.

Accumulation of H_2_O_2 _was accompanied by changes in ROS scavenging enzyme activities [13,14]. Here, we compared salt stress-induced alterations in the activities of the antioxidant enzymes superoxide dismutase (SOD), catalase (CAT) and ascorbate peroxidase (APX) in *OsCYP2 *transgenic and wild-type seedlings. Salt treatment increased the activities of these enzymes in *OsCYP2 *transgenic seedlings to varying degrees (Figure 6C, D and 6E). For wild-type seedlings, CAT activity increased (to a lesser degree than for transgenic seedlings) but the activities of SOD and APX decreased in response to salt stress.

### Expression pattern of *OsCYP2 *in hybrid rice seedlings

**Figure 7:**
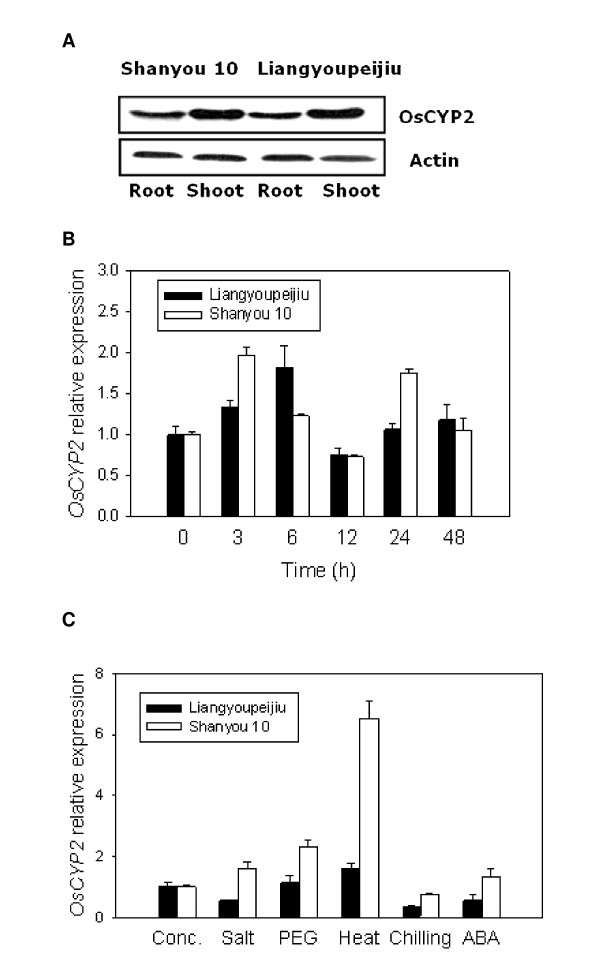
**Expression of *OsCYP2* in hybrid rice seedlings**. **(A) **West blot showed expression of *OsCYP2* in roots and shoots in 10-day-old rice seedlings. **(B) **RT-PCR showed time-course expression of *OsCYP2 *in seedlings of rice hybrid varieties, Shanyou 10 and Liangyoupeijiu, treated with 100 mM NaCl. **(C) **RT-PCR showed expression of *OsCYP2* in hybrid seedlings under various stresses. Conc.: non-treated controls. Salt: 100 mM NaCl at 25°C for 3 h. PEG: 20% (w/v) PEG at 25°C for 3 h. Heat: 45°C for 3 h. Cold treatment: 4°C for 3 h. ABA: 50 mM ABA at 25°C for 3 h. Expression of *OsCYP2* in hybrid rice seedlings was analyzed by RT-PCR. Actin (Os03g0718100) was used as an internal standard.

To better understand *OsCYP2 *function, we utilized RT-PCR to detect temporal and spatial expression patterns of *OsCYP2 *in hybrid rice seedlings. Based on the data in Figure 7A, it appeared that the OsCYP2 expression in roots was less than that in shoots. *OsCYP2 *expression was strongly induced by salt stress (Figure 7B). At different time points (0, 3, 6, 12, 24 and 48 h) after salt treatment (100 mM NaCl), *OsCYP2 *exhibited circadian rhythm expression as time went. Maximal *OsCYP2 *expression occurred at 3 h in Shanyou 10 seedlings and at 6 h in Liangyoupeijiu seedlings, whereas minimal *OsCYP2 *expression occurred at 12 h in Shanyou 10 and Liangyoupeijiu seedlings (Figure 7B). Another peak of *OsCYP2 *expression appeared at 24 h in Shanyou 10 seedlings but not significantly in Liangyoupeijiu seedlings. Interestingly, Shanyou 10 seedlings showed higher maximal *OsCYP2 *expression than Liangyoupeijiu seedlings (Figure 7B). In addition to salt stress, *OsCYP2 *expression was affected by other types of stresses-PEG, heat, cold, or ABA. In Shanyou 10 and Liangyoupeijiu seedlings, *OsCYP2 *expression was induced by PEG and heat but inhibited by cold (Figure 7C). ABA slightly induced expression in Shanyou 10 seedlings but inhibited expression in Liangyoupeijiu seedlings. Generally, Shanyou 10 seedlings showed higher *OsCYP2 *expression than Liangyoupeijiu seedlings under the above mentioned stresses.

## Discussion

The amino acid sequence alignment shows that OsCYP2 is likely to have peptidyl-prolyl cis-trans isomerase (PPIase or rotamase) activity, which catalyzes the cis-trans isomerization of the amide bond between a proline residue and the preceding residue, and functions as a molecular chaperone involved in protein folding, and refolding of denatured proteins. OsCYP2 possesses seven residues, including His-61, Arg-62, Phe-67, Gln-118, Phe-120, Trp-128 and His-33 that show to be associated with PPIase catalysis. Three of these, including His-61, Arg-62 and Phe-120, are most essential for PPIase activity of OsCYP2. The residue Trp-128 is a site binding to cyclosporin A. Seven residues were also found in AtCYP20-2 that had the PPIase activity. In our study, two transgenic lines with *OsCYP2 *overexpression maintain higher levels of total PPIase activity compared to wild type. The addition of CsA is able to reduce total PPIase activity of both wild type and two transgenic lines. Although it has been demonstrated by heterologous expression that OsCYP2 possessed PPIase activity [9], our findings provide powerful evidence at *in vivo *level to validate it.

The mechanisms of plant response or tolerance to salt stress can fall into three categories: tolerance to osmotic stress, Na^+ ^exclusion from leaf blades and tissue tolerance [15]. Osmotic stress response is the first phase that plant responds to salt stress, resulting in the decrease in the rate of leaf growth and rate of photosynthesis. The reduced rate of photosynthesis accelerates the formation of ROS, and increases the activity of enzymes that detoxify ROS [16,17]. These enzymes include SOD, APX, CAT, and the various peroxidases [16,18]. The coordinated activity of the multiple forms of these enzymes in the different cell compartments maintain a balance between the rate of formation and removal of ROS, and control H_2_O_2 _at the levels required for cell signaling. Ionic stresses occur at a later stage, which then leads to senescence of mature leaves. The main site of Na^+ ^toxicity for most plants is the leaf blade, where Na^+ ^accumulates after being deposited in the transpiration stream rather than in the roots [19]. Most Na^+ ^that is transported to the shoot remains in the shoot, because for most plants, the movement of Na^+ ^from the shoot to the roots in the phloem can likely recirculate only a small amount of the Na^+ ^that is transported to the shoot [15]. Therefore, Na^+ ^accumulation in the shoot is dependent on the net delivery of Na^+ ^into the root xylem. Interestingly, several genes that are involved in controlling the net delivery of Na^+ ^into the root xylem have been identified. The plasma membrane Na^+^/H^+ ^antiporter, SOS1, is expressed in stelar cells and could be involved in the efflux of Na^+ ^from stelar cells into the xylem [15]. Meanwhile, SOS1 has also been implicated in retrieval of Na^+ ^from the xylem [20]. Moreover, there is much evidence showing that some members of the high-affinity K^+ ^transporter (*HKT*) gene family play important role in retrieval of Na^+ ^from the xylem. *AtHKT1;1*, a member of *Arabidopsis HKT *gene family, that is involved in the retrieval of Na^+ ^from the xylem before it reaches the shoot [15]. A similar function for the closely related *HKT1;5 *gene family has been identified in rice [3] and wheat [21-23]. Unlike *SOS1 *or members of *HKT *gene family, *OsCYP2*, encodes a rice cyclophilin, inferring that it is likely to function as a molecular chaperone that is involved in protein folding. Over-expression of *OsCYP2 *confers salt tolerance in rice. However, higher leaf or root K^+^/Na^+ ^ratio was not shown in *OsCYP2 *transgenic seedlings under salt stress as compared to wild type (Additional file 5), suggesting that OsCYP2 is not implicated in Na^+ ^accumulation and transport in rice seedlings. Similarly, *OsCYP2 *transgenic seedlings displayed lower free proline level than wild type (Additional file 6), indicating that OsCYP2 does not play a role in osmotic protection of rice seedlings against salt stress. Interestingly, wild-type seedlings exhibited a marked reduction in maximal photochemical efficiency under salt stress, whereas no such change was observed for *OsCYP2*-transgenic seedlings. *OsCYP2*-transgenic seedlings had lower levels of lipid peroxidation products and higher activities of antioxidant enzymes than wild-type seedlings. However, no significant correlations were found between gene expression level and activity level of antioxidant enzymes (Additional file 7, 8). It is suggested that H_2_O_2 _levels are controlled by OsCYP2 up-regulating the activities of SOD, CAT, and APX at post-translation level, not at transcription level, thus resulting in reduced MDA level. This, in turn, protected photosynthesis components of rice leaves against oxidative stress by maintaining the activity of PSII. Therefore, OsCYP2 may be a key regulator that controls ROS level by modulating activities of antioxidant enzymes at translation level.

Here, our results show that OsCYP2 plays a key role in preventing oxidative damage to photosystems. Generally, the two processes that avoid photoinhibition owing to excess light are heat dissipation by the xanthophyll pigments and electron transfer to oxygen acceptors other than water. The latter response necessitates the upregulation of key enzymes for regulating ROS levels such as SOD, APX, CAT, and the various peroxidases [16,18]. Obviously, the above knowledge leads us to infer that OsCYP2 may be implicated in the process of electron transfer to oxygen acceptors. However, sufficient evidence is still lacking, further studies are needed to address this possibility.

In this study, *OsCYP2 *expression is induced by salt stress. Interestingly, *OsCYP2 *shows circadian rhythm expression as time goes. As a result, we speculate that response of *OsCYP2 *to salt stress is likely to be regulated by circadian rhythm. Moreover, circadian rhythm expression of *OsCYP2 *in Shanyou 10, a salt-tolerant hybrid variety, occurs earlier than that in Liangyoupeijiu, a salt-sensitive hybrid variety, suggesting earlier response of *OsCYP2 *to salt stress is likely to be associated with salt tolerance of rice seedlings. In addition to salt stress, *OsCYP2 *expression is affected by other types of stresses-PEG, heat, or ABA induced expression in Shanyou 10 seedlings but inhibited expression in Liangyoupeijiu seedlings. In addition, cold stress inhibits *OsCYP2 *expression in Shanyou 10 and Liangyoupeijiu seedlings.These data suggest that OsCYP2 expression is not specific in salt stress, but is ubiquitous in the response of rice seedlings to other types of stresses, including drought, heat and cold. Importantly, the above conclusion is consistent with the previous findings that *OsCYP2 *can respond to various stresses including high salt, drought, heat, oxidative stress and hypoxia stress [9,24]. Therefore, we speculate that OsCYP2 may function as a key integrator in response to multiple stresses.

## Conclusions

Comparative proteomics identified a rice cyclophilin, OsCYP2 that is up-regulated during salt-induced stress. Over-expression of OsCYP2 confers salt tolerance in rice. Under salt stress, OsCYP2 is likely to up-regulate the activities of antioxidant enzymes (SOD, CAT, and APX) at post-translation level to control H_2_O_2 _levels, resulting in reduced MDA levels, which may prevent oxidative damage to photosystems. Unfortunately, OsCYP2 is not implicated in Na^+ ^accumulation and transport and osmotic protection in rice seedlings. OsCYP2 expression is not only induced by salt stress, but also regulated by circadian rhythm. Moreover, OsCYP2 is also likely to act as a key component that is involved in signal pathways of other type of stresses-PEG, heat, cold, or ABA.

## Methods

### Plant material and salt treatment

Seeds of Shanyou 10 and Liangyoupeijiu were supplied by the Wu Wang Nong Seed Group (Hangzhou, Zhejiang province, China). Four replicates of 50 seeds for each treatment of each genotype were placed in germination boxes (18 cm × 13 cm × 10 cm) containing two layers of moistened blotters with 10 ml of 100 mM NaCl. The seeds were germinated for 10 days at 25°C. The NaCl solution was changed every day to maintain a constant concentration of NaCl.

### Relative biomass and length of rice seedlings

Four replicates of 10 fresh shoots or 10 fresh roots of 10-day-old seedlings for each treatment of each genotype were weighed. These shoots or roots were then oven dried at 70°C until they reached a constant dry weight (DW) [25]. The lengths of 10 shoots or 10 roots from the four replicates of 10-day-old seedlings for each treatment of each cultivar were measured. The distance from the basal part of shoot to tip of the longest leaf was calculated as the length of seedling. The standard error (SE) on the mean fresh weight (FW), dry weight (DW) or length of shoot and root was calculated. The percentage of relative FW, DW, or shoot/root length of the salt treated samples was calculated in relation to non-treated.

### Protein extraction

The shoots of 10-day-old rice seedlings were harvested. 1 g FW of shoots were ground in liquid nitrogen and suspended in 5 ml of 10% (w/v) trichloroacetic acid in acetone with 0.07% (w/v) β-mercaptoethanol at -20°C for 1 h, followed by centrifugation for 15 min at 35000 × *g*. The pellets were resuspended in acetone with 0.07% (w/v) β-mercaptoethanol and incubated at -20°C for 1 h and then centrifuged for 15 min at 4°C. This step was repeated three times, and the pellets were lyophilized. The crude protein power was solubilized in lysis buffer (8 M urea, 2 M thiourea, 4% CHAPS, 0.5% Ampholine (pH 3-10), 50 mM DTT, and 1 mM PMSF) for 1 h at room temperature, followed by centrifugation for 15 min at 15000 × *g*. The supernatant was collected in a 1.5-ml tube, and a 40 μl sample was taken to determine the protein concentration. Protein concentration was determined using the Bradford assay with bovine serum albumin as the standard.

### 2-D electrophoresis analysis

For analytical and preparative gels of 2-DE, 120 μg and 300 μg of shoot proteins were loaded onto a single IPG gel strip (170 mm, pH 5-8 or pH 7-10, Bio-Rad, USA), respectively. IEF was carried out using the PROTEAN IEF system (Bio-Rad). IPG strips were rehydrated in rehydration buffer (8M urea, 2% (w/v) CHAPS, 0.5% (v/v) Ampholine (pH 3-10), 50 mM DTT and protein samples) for 12 h at 50 V. IEF was performed in three steps: 250 V for 15 min, 10000 V for 5 h and then for a total of 60000 Vh at 10000 V. The gel strips were equilibrated in two steps: 6 ml equilibration buffer I (6 M urea, 2% SDS, 0.375 M Tris-HCl pH 8.8, 20% (v/v) glycerol and 130 mM DTT) for 10 min and 6 ml equilibration buffer II (buffer I lacking DTT but containing 135 mM iodoacetamide) for 10 min.

### Image and data analysis

Silver-stained gels were scanned using a Microtek 6180 scanner at a resolution of 600 dots per inch (dpi), and data were analyzed using PDQuest 8.0 software (Bio-Rad). Specifically, gel image filter, spot detection, background subtraction and spot matching were performed. Prior to spot matching among gel images, one gel image was selected as a reference. After automatic matching, the unmatched spots of the member gels were added to the reference gel. The area of each spot was defined as the sum of the intensities of all pixels that made up the spot. To compare quantitative variations in intensity of protein spots, the spot areas were normalized as a percentage of the total area in all of the spots present in the gel. The resulting data from image analysis were transferred to PDQuest 8.0 software for query protein spots showing quantitative or qualitative variations.

### In-gel digestion, MALDI-TOF MS, and ESI-MS/MS analysis

In-gel digestion of proteins was performed as described [26] with some modifications. Protein spots were excised from the preparative gels and washed twice with Milli-Q water, and then were destained twice for 5 min with 200 μl of freshly prepared equi-volume solution of 100 mM Na_2_S_2_O_3 _and 30 mM K_3_Fe(CN)_6_. The samples were washed twice for 5 min with Milli-Q water and were cut into pieces, dried in a vacuum system, and then digested overnight at 37°C with 10 μg/ml sequencing-grade modified trypsin (Roche, Germany) in 25 mM NH_4_HCO_3_. The peptide mixtures were extracted with 40 μl 0.5% trifluoroacetic acid for 1 h at 40°C, 40 μl 0.25% trifluoroacetic acid in 50% ACN for 1 h at 30°C, and 25 μl ACN for 5 min, respectively. All three extracts for each sample were combined and lyophilized. The resulting lyophilized tryptic peptides were dissolved in 5 mg/ml α-cyano-4-hydroxycinnamic acid (CHCA) containing 50% ACN and 0.1% trifluoroacetic acid. MS analysis of tryptic peptides was performed using a MALDI-TOF mass spectrometer (reflex; Bruker Daltonics, Germany). The peptide calibration standard "mono" (Bruker Daltonics) was used for internal calibration to ensure the accuracy of protein identification. Masslynx software (version 3.5) was used for peak-picking. The S/N of peaks was set at three. Peaks below 1000 m/z were ignored. Mass range was from 1000 up to 4000 m/z. Resolution was set at 10000. The PMF data were analyzed using MASCOT searching tools (version 1.9, http://www.matrixscience.com  Matrix Science, London, UK). NCBInr (version 20050513) and rice were selected as the database and taxonomy, respectively. All peptide masses were assumed to be monoisotopic and [M+H]^+^. Modifications of carbamidomethylation and oxidation were considered. The mass accuracy was set at ± 100 ppm, and the maximum number of missed cleavages was set at one. The identified protein had to have the top MASCOT score and ≥ 4 matched peptides. The coverage of the protein by the matching peptides was > 10%. Protein scores that were obtained from the analysis with Mascot software indicated the probability of a true positive identification (p < 0.05) and must be at least 60.

Several tryptic peptides of interest were analyzed using ESI MS/MS. Raw MS/MS data were processed using MaxEnt software (version 3) which performed peak list geneation. MASCOT search engine (version 1.9, http://www.matrixscience.com; Matrix Science, London, UK) was used for all MS/MS ions search. NCBInr (version 20050513) and rice were selected as the database and taxonomy, respectively. All peptide masses were assumed to be monoisotopic and [M+H]^+^. Cysteine carbamidomethylation and methionine oxidation were selected as variable modifications. One missing cleavage was allowed. Precursor error tolerance was set to <0.2 Da and MS/MS fragment error tolerance < 0.2 Da. The identified protein should have at least two peptides matched and individual ions scores greater than 38 with expected value < 0.05. Maximal number of protein entries was set at five. Cut-off score for accepting individual MS/MS spectra was set at zero.

### Gene cloning and transformation

The *OsCYP2 *coding region was obtained by RT-PCR amplification with the following primers: *OsCYP2*-F, 5'-TCTAGAATGTCGAACACGAGGGTGTT-3'; *OsCYP2*-R, 5'-GGTACCCTAGGAGAGCTGGCCGCAGT-3'. PCR products were recovered with a glass milk kit (BioDev Company, Beijing, China). Recovered fragments were ligated into the PMD18-T vector (TaKaRa, Japan). The ligated products were then transformed into *E. coli *DH5α competent cells (TaKaRa, Japan). Twenty positive colonies were selected and identified by PCR. The positive plasmids extracted from these colonies were digested with EcoRI or PstI (TaKaRa, Japan) to confirm that target fragments were inserted into PMD18-T, and then positive plasmids were sequenced using an ABI 3700 (ABI, USA).

Each correct PCR fragment was digested with XbaI and KpnI and inserted into the binary vector pCAMBIA1300-based super promoter [27]. *Agrobacterium *strain EHA105 was introduced into rice (*O. sativa *cv. Aichi ashahi) using *Agrobacterium*-mediated transformation [28,29]. A total of 120 lines of hygromycin- resistant (1 μg/ml) transgenic plants were selected, and their T3 plants were analyzed for phenotypic changes under salt stress.

### Phenotypic analysis of *OsCYP2 *transgenic seedlings under salt stress

Three replicates of 50 T3-transgenic rice seeds with a single copy insertion of hygr^+ ^(Additional file 3) or wild-type seeds were placed in germination boxes (18 cm × 13 cm × 10 cm) containing two layers of blotters moistened with distilled water. The boxes were then transferred to the chamber, and seeds were germinated at 25°C under 16 h light/8 h dark at an illumination intensity of 60 mmol m^-2 ^s^-1 ^for 10 days. Transgenic and wild-type seedlings were transferred to germination boxes (18 cm × 13 cm × 10 cm) containing two layers of blotters moistened with 200 mM NaCl solution or distilled water. After 5 days, the phenotypes of seedlings were observed and photographed using a digital camera.

### Assay of maximal photochemical efficiency (Fv/Fm)

Chlorophyll fluorescence was determined with chlorophyll fluorescence imaging system (IMAGING PAM; Heinz Walz, Effeltrich, Germany). To measure the maximal quantum efficiency of PSII (Fv/Fm), rice seedlings were dark-adapted for 30 min. The measured light intensity for normal light and saturating light were 1 and 10, respectively. Fv/Fm was also measured by FMS-2 pulse amplitude fluorimeter (Hansatech Instruments Ltd., Kings Lynn, Norfolk, UK). Rice seedlings were maintained in darkness for 30 min before measurement of Fv/Fm. Minimal fluorescence (Fo) was measured under a weak pulse of modulating light over a 0.8-s period, and maximal fluorescence (Fm) was obtained after a saturating pulse of 0.7 s at 8000 μmol m^-2 ^s^-1^.

### Antioxidant enzyme extraction and activity assay

For the enzyme assays, 0.5 g FW of shoots were ground with 3 ml of ice-cold 25 mM sodium phosphate buffer (pH 7.8) containing 0.2 mM EDTA, 2 mM ascorbate acid (AsA) and 2% (w/v) polyvinylpyrrolidone. The suspensions were centrifuged at 4°C for 20 min at 12,000 × *g*, and the resulting supernatants were used to determine enzymatic activity. Superoxide dismutase (SOD) activity was assayed by measuring the ability to inhibit the photochemical reduction of nitroblue tetrazolium (NBT) as described [30]. Catalase (CAT) activity was measured as the decline in absorbance at 240 nm due to the decrease of extinction of H_2_O_2 _as described [31]. Ascorbate peroxidase (APX) was measured by the decrease in absorbance at 290 nm as described [32].

### Determination of MDA and H_2_O_2 _content in rice shoots

Lipid peroxidation was measured as the amount of malondialdehyde (MDA) determined by the TBA reaction as described [33]. Shoot samples (0.5 g) were homogenized in 3 ml of 50 mM PBS (pH 7.8) containing 2% (v/v) polyvinylpyrrolidone, and then centrifuged at 15,000 × *g *for 20 min. Three ml of 10% (w/v) trichloroacetic acid containing 0.6% (w/v) TBA was added to 1 ml of the supernatant aliquot. The mixture was heated at 100°C for 30 min and then quickly cooled in an ice bath. The mixtures were centrifuged at 10,000 × *g *for 10 min, and their absorbance was measured at 532 nm. The value for non-specific absorption at 600 nm was subtracted from the 532 nm reading. The MDA content was calculated using its extinction coefficient of 155 mM^-1 ^cm^-1 ^and expressed as μmol g^-1 ^FW.

Plant tissue H_2_O_2 _was extracted by cold acetone as described [33]. Shoots (1 g FW) were homogenized in 5 ml cold acetone and then centrifuged at 10,000 × *g *for 10 min. To 1 ml of the supernatant aliquot, 3 ml of extract containing a 3:1 (v/v) ratio of CCl_4 _and CHCl_3 _and 5 ml of distilled water were added and mixed successively, and then centrifuged at 4,000 × *g *for 1 min. The supernatant was removed and tested for H_2_O_2_. H_2_O_2 _content was determined by ferric-xylenol orange complex as described [34].

### Peptidyl prolyl cis-trans activity assay

Rice shoots (0.3 g FW) were homogenized in 50 mM Tris-HCl (pH 8.0) containing 1 mM EDTA, and centrifuged at 13000 × g for 15 min at 4°C. 50 μl supernatant was used for peptidyl prolyl cis-trans isomerase (PPIase) activity. PPIase activity was assayed in a coupled reaction with chymotrypsin, as described^9 ^with some modifications. The assays were performed at 0°C for 120 s. The 1 ml assay mixture contained 80 μM N-succinyl-ala-ala-prophe-p-nitroanilidine (Sigma, USA) as test peptide and assay buffer [50 mM HEPES (pH 8.0), 150 mM NaCl, 0.05% Triton X-100], The reaction was initiated by the addition of 100 μl chymotrypsin (5 mg/ml) (Sigma, USA) and the change in absorbance at 390 nm was monitored using a spectrophotometer (Shimadzu UV2550). Cyclophilin associated PPIase activities were determined by the extent of inhibition of reaction in the presence of cyclosporin A (Sigma, USA). The inhibitor was added to the assay mix 30 min before the start of the reaction and incubated at 4°C.

### Real-time PCR

Frozen leaf tissue was homogenized in liquid nitrogen using a mortar and pestle. Total RNA was extracted using Trizol according to the supplier's recommendation (Invitrogen, Karlsruhe, Germany). Residual DNA was removed with an RNase-free DNase (Fermentas, EU). One microgram total RNA was reverse-transcribed using 0.5 μg of Oligo (dT) 20 and 200 units of ReverTra Ace (TOYOBO, Japan) following the supplier's recommendation. Quantitative real time PCR was performed using the Opticon 2 Real-time PCR Detection System (Bio-Rad, Hercules, CA, USA). PCRs were performed using the SYBR Green Supermix (Bio-Rad). The PCR conditions consisted of 40 cycles of denaturation at 95°C for 30 s, annealing at 59°C for 45 s and extension at 72°C for 30 s. A dissociation curve was generated at the end of each PCR cycle to verify that a single product was amplified using software provided with the Opticon 2 Real-time PCR Detection System. To minimize sample variations, mRNA expression of the target gene was normalized relative to the expression of the housekeeping gene actin. All experiments were repeated three times for cDNA prepared for two samples. The quantification of mRNA levels is based on the method of Livak and Schmittgen (2001). The threshold cycle (Ct) value of actin (*Os03g0718100*) as internal standard was subtracted from that of the gene of interest to obtain a ΔCt value. The Ct value of untreated control sample was subtracted from the ΔCt value to obtain a ΔΔCt value. The fold changes in expression level relative to the control were expressed as 2^-ΔΔ^Ct. The following primers were designed for gene-specific transcript amplification: *OsCYP2-F*: 5'-GCCTTTCGCCAGTATCAGTC-3', *OsCYP2-R*: 5'-CAGATCCAACTCCACCGAAT-3'; Actin-F: 5'-GACCTTGCTGGGCGTGAT-3', Actin-R: 5'-GTCATAGTCCAGGGCGATGT-3'.

### Preparation of antiserum and western blot analysis

According to the OsCYP2 amino acid sequence, the peptide fragment 44-KGVGKSGKPLHYKG-57 was determined as a protein-surface antigen. The peptide containing an additional N-terminal cysteine was synthesized and purified on a resin. The purified peptide was used to raise polyclonal antibodies in rabbits (HuaAn Biotechnology Co., Ltd., Hangzhou, China).

Rice shoots (0.5 g FW) were ground into a fine powder in liquid nitrogen and then added to 2 ml of protein extraction buffer containing 50 mM Tris-HCl, pH 8, 1 mM EDTA, 10 mM NaCl, 1% SDS, 0.5% (v/v) 2-mercaptoethanol, 0.1 mM PMSF, 0.1 mM DTT and 0.1% (v/v) Triton X-100, and ground until shoot power was well homogenized. The mixtures were centrifuged at 4°C for 15 min at 14,000 × *g*, and the supernatant was transferred into a 5-ml centrifuge tube. The protein concentration was determined using the *RC DC *protein assay kit II (Bio-Rad, USA). Total protein (20 μg) from each sample was subjected to electrophoresis on a 15% SDS-PAGE gel. Proteins in the gel were transferred to PVDF membranes by an electric transforming system, and the membranes were blocked with 5% (w/v) skim milk. The blot was incubated with the rabbit antiserum raised against OsCYP2 diluted 1:1000 in TBST containing 25 mM Tris base pH 8.0, 140 mM NaCl, 3 mM KCl and 0.05% (v/v) Tween 20 for 1 h and washed three times for 5 min each in TBST (Tris-buffered saline/Tween 20). The blot was then probed with the secondary antibody (HRP-labeled goat anti-rabbit IgG (H+L)) diluted 1:5000, and the reactive band was visualized using ECL (Multiscience Biotech Co., Ltd., Hangzhou, China).

### Statistical analysis

The experimental design was set up with genotypes as main plots and treatments as subplots. The treatment combinations were completely randomized with respect to the placement of the germination boxes in a germination chamber with five layers of rack for each genotype. Three replications of the experiment were conducted on different dates. Analyses of variance (ANOVA) were conducted by Duncan's multiple range test. Before analysis of variance, percentages were transformed according to y = arcsin[sqr(x/100)]. All data were analyzed according to a factorial model and replicates as random effects. Means were compared among treatments by LSD (least significant difference) at 0.05 confidence level.

## Abbreviations

ABA: abscissic acid; APX: ascorbate peroxidase; CAT: catalase; cDNA: complementary DNA; CHAPS: 3[(cholamidopropy1) dimethylammonio]-1-propane sulphonate; CsA: cyclosporin A; 2-D: two-dimensional; 2-DE: two-dimensionsl polyacrylmide gel electrophoresis; DTT: dithiothreitol; DW: dry weight; EDTA: ethylenediaminetetraacetic acid; ESI-MS: electrospray Ionization mass spectrometry; FW: fresh weight; IEF: isoelectric focusing; IPG: immobilized pH gradient; MALDI-TOF: matrix-assisted laser desorption-ionization time-of-flight; MDA: malonaldehyde; MS: mass spectrometry; MW: molecular weight; NBT: nitroblue tetrazolium; PAGE: polyacrylamide gel electrophoresis; PBS: phosphate buffer solution; PCR: polymerase chain reaction; PEG: polyethylene glycerol; PMF: peptide mass fingerprint; PMSF: phenylmethlsulfonyl fluoride; PPIase: peptidyl prolyl cis-trans isomerase; PSII: photosystem II; PVDF: polyvinylidend difluoride; PVP: Polyvinylpyrrolidone; QTL: quantitative trait loci; ROS: reactive oxygen species; SDS: sodium dodecyl sulfate; SE: standard error; SOD: superoxide dismutase; *SOS1*: salt overly sensitive1; TBA: thiobarbituric acid; TCA: trichloroacetic acid.

## Authors' contributions

SLR carried out 2-DE analysis, conceived of the study, participated in its design and coordination and completed the manuscript. HSM carried out physiological analysis and participated in the design of the study. SHW carried out phenotypic analysis. YPF carried out gene cloning and transformation. YX participated in gene cloning and construction of binary vector. WZL carried out western blot analysis and participated in the sequence alignment. FW carried out RT-PCR analysis and biochemical assays. JXT participated in physiological analysis. SZW participated in gene transformation. HZC participated in phenotype identification and statistical analysis. All authors read and approved the final manuscript.

## Supplementary Material

Additional file 1 **OsCYP2 (p8 protein spot) was identified by MALDI-TOF-MS and ESI-MS/MS**. (A) PMF map of OsCYP2 (p8 protein) digested with trypsin. (B), (C) Sequences of two peptide fragments (m/z 1424.64 and 1656. 64) with asterisk (*) from the PMF map were analyzed using ESI-MS/MS. Click here for file

Additional file 2 **Identification of rice leaf proteins by ESI-MS/MS**. Click here for file

Additional file 3 **Genetic analysis of *CYP2 *transgenic lines (T1 generation) containing a hygromycin marker**. Click here for file

Additional file 4 **Phenotypes of rice seedlings under salt stress**. *OsCYP2 *transgenic rice lines showed salt tolerant phenotypes. Three-week-old rice seedlings were treated with 150 mM NaCl under water culture condition. After 7 days, phenotypes of rice seedlings were observed. WT represents the wild-type seedling, *Aichi ashahi *that was used as a reference rice cultivar. (A) WT and OE1 (overexpressed line no.). (B) WT and OE2 (overexpressed line no.). Click here for file

Additional file 5 **The ratio of potassium (K) to sodium (Na) of rice seedlings under salt stress**. Three-week-old rice seedlings were treated for 2 d with 150 mM NaCl under water culture condition. The total potassium or sodium content of rice shoots or roots was determined using atomic absorption spectroscopy, respectively. (A) Shoots. (B) Roots. Click here for file

Additional file 6 **The free proline content of rice seedlings under salt stress**. Three-week-old rice seedlings were treated for 2 d with 150 mM NaCl under water culture condition. The free proline content of rice shoots was determined by ninhydrin reaction. Click here for file

Additional file 7 **Expression pattern of antioxidant enzyme genes in transgenic rice seedling under salt stress**. Ten-old rice seedlings were treated for 1 d with 200 mM NaCl. Expression of several genes was quantified using real time PCR. (A) *Gu/Zn- SOD *(accession no. D01000.1) (B) *Mn- SOD *(accession no. L19436.1) (C) *Fe- SOD *(accession no. AY770495.1) (D) Os*Cat *(accession no. AY339372.1) (E) Os*CatC *(accession no. AB020502) (F) *mAPX *(accession no. AY382617.1) (G) *cAPX *(accession no. AY254495.1) (H) *sAPX *(accession no. AB114855). The housekeeping gene, Actin (*Os03g0718100*) was used as internal standard. Click here for file

Additional file 8 **Correlations between activities of antioxidant enzymes and expression of corresponding genes**. Click here for file
