# Supplementary material for: Proteomic identification of OsCYP2, a rice cyclophilin that confers salt tolerance in rice (Oryza sativa L.) seedlings when overexpressed
Source: BMC Plant Biol. 2011 Feb 16;11:34. doi: 10.1186/1471-2229-11-34 (PMC3050798; doi:10.1186/1471-2229-11-34)
Supplement: Additional file 1 — OsCYP2 (p8 protein spot) was identified by MALDI-TOF-MS and ESI-MS/MS. (A) PMF map of OsCYP2 (p8 protein) digested with trypsin. (B), (C) Sequences of two peptide fragments (m/z 1424.64 and 1656. 64) with asterisk (*) from the PMF map were analyzed using ESI-MS/MS. [file 1471-2229-11-34-S1.DOC]

**A** PMF map of OsCYP2 （p8 protein）digested with trypsin


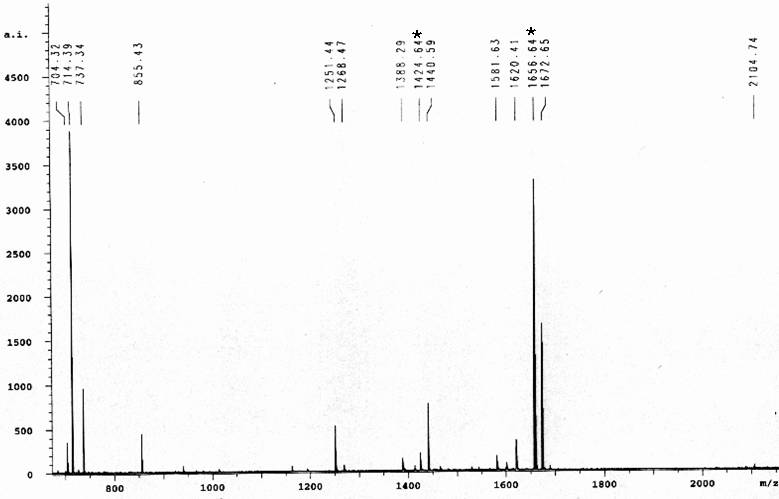


**B**

MS/MS Fragmentation of **VIPEFMCQGGDFTR**

Found in **AAA57046**, [*Oryza sativa* (japonica cultivar-group)]


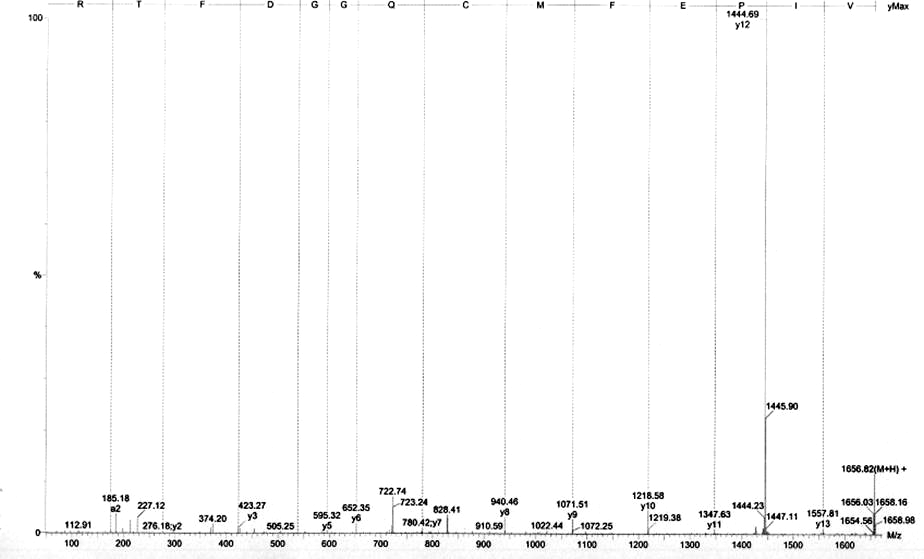


**C**

MS/MS Fragmentation of **VFFDMTVGGAPAGR**

Found in **AAA57046**, [*Oryza sativa* (japonica cultivar-group)]


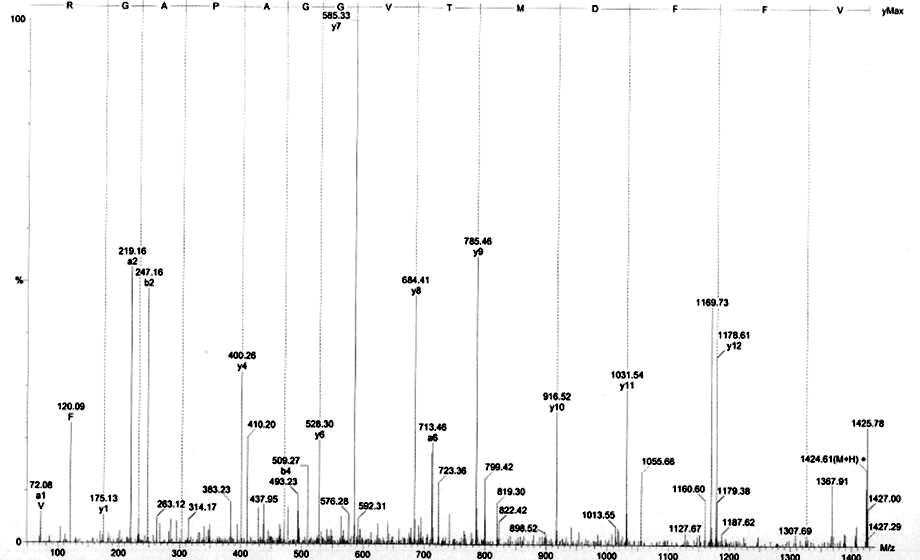


**Figure S1. OsCYP2 (p8 protein spot) was identified by MALDI-TOF-MS and ESI-MS/MS.** (A) PMF map of OsCYP2 （p8 protein） digested with trypsin. (B), (C) Sequences of two peptide fragments (m/z 1424.64 and 1656. 64) with asterisk (*) from the PMF map were analyzed using ESI-MS/MS.
