# Supplementary material for: Proteomic identification of OsCYP2, a rice cyclophilin that confers salt tolerance in rice (Oryza sativa L.) seedlings when overexpressed
Source: BMC Plant Biol. 2011 Feb 16;11:34. doi: 10.1186/1471-2229-11-34 (PMC3050798; doi:10.1186/1471-2229-11-34)
Supplement: Additional file 2 — Identification of rice leaf proteins by ESI-MS/MS. [file 1471-2229-11-34-S2.DOC]

**Table S2. Identification of rice leaf proteins by ESI-MS/MS**

| Spot no.^a^ | Protein name | Match peptide sequences | Modifications | Ion score | Ion score for acceptance ^c^ |
| --- | --- | --- | --- | --- | --- |
| P1 | Putative glutathione S-transferase | LLGVWSSPYAIR | None ^b^ | 52 | 38 |
|  |  | SLPYEYVEENLGDK | None | 57 | 38 |
|  |  | TPALAAWEER | None | 44 | 38 |
| P2 | Manganese superoxide dismutase | KHHATYVANYNKA | None | 52 | 38 |
|  |  | KLGWAIDEDFGSFEALVKK | None | 60 | 38 |
|  |  | KNVRPDYLSNIWKV | None | 45 | 38 |
| P3 | dehydroascorbate reductase | LIDVQNKPDWFLK | None | 51 | 38 |
|  |  | ALLTELQALEEHLK | None | 45 | 38 |
|  |  | AHGPFINGQNISAADLSLAPK | None | 49 | 38 |
| P4 | putative phosphogluconate dehydrogenase | KVEGNLPVYGFHDPASFVNSIQKPRV | None | 47 | 38 |
|  |  | KWTVQQAAELSVAAPTIEASLDSRF | None | 53 | 38 |
|  |  | RDYFGAHTYERV | None | 49 | 38 |
| P5 | Putative L-aspartate oxidase | CAQVHISSIPHFIGAK | None | 49 | 38 |
|  |  | EGGHSHNRIVHSADMTGR | None | 43 | 38 |
|  |  | VLAHFPNIAAECLR | None | 45 | 38 |
| P6 | putative cold shock protein-1 | KGFGFITPDDGGEDLFVHQSSLKS | None | 62 | 38 |
|  |  | RGYGGGGGGYGGGDRG | None | 55 | 38 |
|  |  | RDCSQGGGGGGGYGGGGGGYRG | Carbamidomethyl (C) | 48 | 38 |
| P7 | Prohibitin | TRPHNFSSNSGTK | None | 52 | 38 |
|  |  | LISEATAAAGTGLIELR | None | 48 | 38 |
|  |  | SPNVAYVPAGDNGR | None | 62 | 38 |
| P9 | Putative oxygen -evolving enhancer protein 3-1 | DTDLPLRER | None | 56 | 38 |
|  |  | FYLQPLPPAEAAAR | None | 64 | 38 |
|  |  | QWPFVRDDLR | None | 61 | 38 |

^a^ Spot Nos refer to spot number. ^b^ None: without modifications. ^c^ Ion score for acceptance: individual ion score > 38 indicate identity or extensive homology (p < 0.05).
