# Supplementary material for: Proteomic identification of OsCYP2, a rice cyclophilin that confers salt tolerance in rice (Oryza sativa L.) seedlings when overexpressed
Source: BMC Plant Biol. 2011 Feb 16;11:34. doi: 10.1186/1471-2229-11-34 (PMC3050798; doi:10.1186/1471-2229-11-34)
Supplement: Additional file 3 — Genetic analysis of CYP2 transgenic lines (T1 generation) containing a hygromycin marker. [file 1471-2229-11-34-S3.DOC]

**Table S1. Genetic analysis of *CYP2* transgenic lines (T1 generation) containing a** hygromycin marker

| Line no. | Seedlings with hygr^+^ | Seedlings without hygr^+^ | χ^2^ value  (3:1) | χ^2^ value  (P < 0.05) |
| --- | --- | --- | --- | --- |
| OE1 | 30 | 8 | 0.14 | 3.84 |
| OE2 | 26 | 11 | 0.23 |
