# Supplementary material for: Proteomic identification of OsCYP2, a rice cyclophilin that confers salt tolerance in rice (Oryza sativa L.) seedlings when overexpressed
Source: BMC Plant Biol. 2011 Feb 16;11:34. doi: 10.1186/1471-2229-11-34 (PMC3050798; doi:10.1186/1471-2229-11-34)
Supplement: Additional file 4 — Phenotypes of rice seedlings under salt stress. OsCYP2 transgenic rice lines showed salt tolerant phenotypes. Three-week-old rice seedlings were treated with 150 mM NaCl under water culture condition. After 7 days, phenotypes of rice seedlings were observed. WT represents the wild-type seedling, Aichi ashahi that was used as a reference rice cultivar. (A) WT and OE1 (overexpressed line no.). (B) WT and OE2 (overexpressed line no.). [file 1471-2229-11-34-S4.DOC]

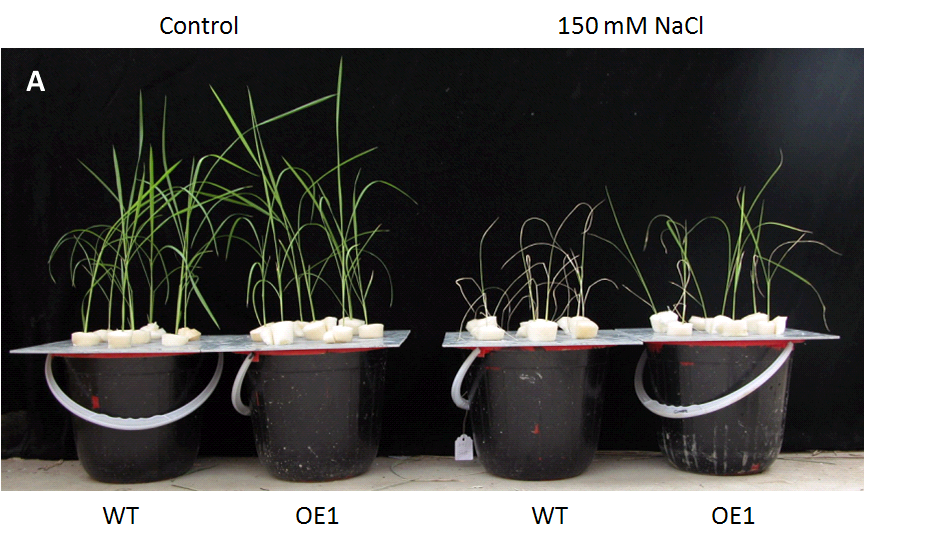


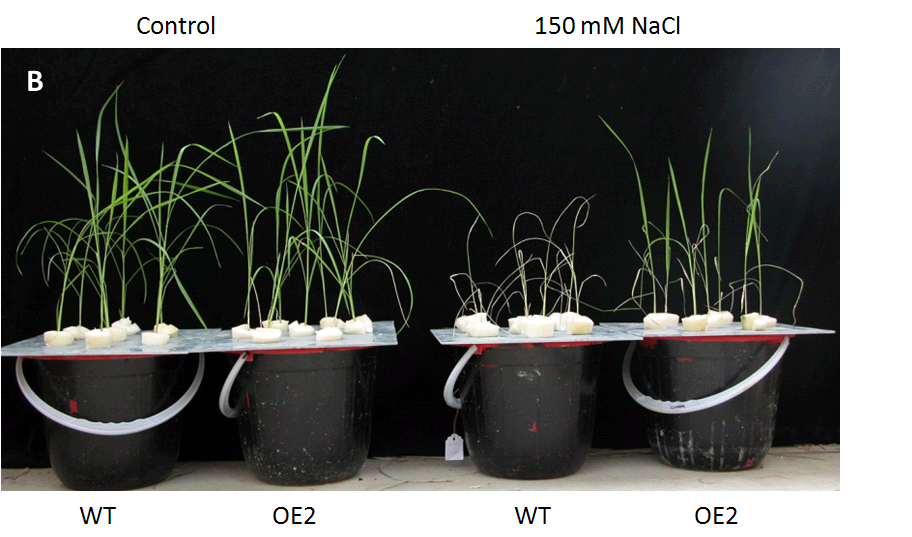


**Figure S2. Phenotypes of rice seedlings under salt stress.** *OsCYP2* transgenic rice lines showed salt tolerant phenotypes. Three-week-old rice seedlings were treated with 150 mM NaCl under water culture condition. After 7 days, phenotypes of rice seedlings were observed. WT represents the wild-type seedling, *Aichi ashahi* that was used as a reference rice cultivar. (A) WT and OE1 (overexpressed line no.). (B) WT and OE2 (overexpressed line no.).
