# Supplementary material for: Proteomic identification of OsCYP2, a rice cyclophilin that confers salt tolerance in rice (Oryza sativa L.) seedlings when overexpressed
Source: BMC Plant Biol. 2011 Feb 16;11:34. doi: 10.1186/1471-2229-11-34 (PMC3050798; doi:10.1186/1471-2229-11-34)
Supplement: Additional file 5 — The ratio of potassium (K) to sodium (Na) of rice seedlings under salt stress. Three-week-old rice seedlings were treated for 2 d with 150 mM NaCl under water culture condition. The total potassium or sodium content of rice shoots or roots was determined using atomic absorption spectroscopy, respectively. (A) Shoots. (B) Roots. [file 1471-2229-11-34-S5.DOC]

A

B

**Figure S3. The ratio of potassium (K) to sodium (Na) of rice seedlings under salt stress.** Three-week-old rice seedlings were treated for 2 d with 150 mM NaCl under water culture condition. The total potassium or sodium content of rice shoots or roots was determined using atomic absorption spectroscopy, respectively. (A) Shoots. (B) Roots.
