# Supplementary material for: Proteomic identification of OsCYP2, a rice cyclophilin that confers salt tolerance in rice (Oryza sativa L.) seedlings when overexpressed
Source: BMC Plant Biol. 2011 Feb 16;11:34. doi: 10.1186/1471-2229-11-34 (PMC3050798; doi:10.1186/1471-2229-11-34)
Supplement: Additional file 6 — The free proline content of rice seedlings under salt stress. Three-week-old rice seedlings were treated for 2 d with 150 mM NaCl under water culture condition. The free proline content of rice shoots was determined by ninhydrin reaction. [file 1471-2229-11-34-S6.DOC]

**Figure S4. The free proline content of rice seedlings under salt stress.** Three-week-old rice seedlings were treated for 2 d with 150 mM NaCl under water culture condition. The free proline content of rice shoots was determined by ninhydrin reaction.
