# Supplementary material for: Proteomic identification of OsCYP2, a rice cyclophilin that confers salt tolerance in rice (Oryza sativa L.) seedlings when overexpressed
Source: BMC Plant Biol. 2011 Feb 16;11:34. doi: 10.1186/1471-2229-11-34 (PMC3050798; doi:10.1186/1471-2229-11-34)
Supplement: Additional file 7 — Expression pattern of antioxidant enzyme genes in transgenic rice seedling under salt stress. Ten-old rice seedlings were treated for 1 d with 200 mM NaCl. Expression of several genes was quantified using real time PCR. (A) Gu/Zn- SOD (accession no. D01000.1) (B) Mn- SOD (accession no. L19436.1) (C) Fe- SOD (accession no. AY770495.1) (D) OsCat (accession no. AY339372.1) (E) OsCatC (accession no. AB020502) (F) mAPX (accession no. AY382617.1) (G) cAPX (accession no. AY254495.1) (H) sAPX (accession no. AB114855). The housekeeping gene, Actin (Os03g0718100) was used as internal standard. [file 1471-2229-11-34-S7.DOC]

A

B

C

D

E

F

G

H

**Figure S5. Expression pattern of antioxidant enzyme genes in transgenic rice seedling under salt stress.** Ten-old rice seedlings were treated for 1 d with 200 mM NaCl. Expression of several genes was quantified using real time PCR. (A) *Gu/Zn- SOD* (accession no. D01000.1) (B) *Mn- SOD* (accession no. L19436.1) (C) *Fe- SOD* (accession no. AY770495.1) (D) Os*Cat* (accession no. AY339372.1) (E) Os*CatC* (accession no. AB020502) (F) *mAPX* (accession no. AY382617.1) (G) *cAPX* (accession no. AY254495.1) (H) *sAPX* (accession no. AB114855). The housekeeping gene, Actin (*Os03g0718100*) was used as internal standard. The following primers were designed for gene-specific transcript amplification: *Cu/Zn-SOD-F*: 5’- GAAGCACCACGCCACCTAC -3’, *Cu/Zn-SOD-R*: 5’- ACCACCACCCTCGCTGATA -3’; *Mn-SOD-F*: 5’-GAAGCACCACGCCACCTAC-3’, *Mn-SOD-R*: 5’-ACCACCACCCTCGCTGATA-3’; *Fe-SOD-F*: 5’-TACGGCGGAGTAGTGAGA-3’, *Fe-SOD-R*: 5’- GGCAGAGGAAAGAGGAGA -3’; Os*Cat*-F: 5’- AGTTTGACAGGGAGCGTAT-3’, Os*Cat*-R: 5’- GGTCTGAACACCAGGAGC-3’; Os*CatC*-F: 5’-AAGCCGAGCATGTAAGGAGA-3’, Os*CatC* -R: 5’- ACACGAATTGTGCGGTGATA-3’; *mAPX*-F: 5’- GAAACAGAGGCATCCCAAGA -3’, *mAPX*-R: 5’- CCAGGCACCATCAAATCC -3’; *cAPX*-F: 5’- ATACCCACCATCTCCTACGC -3’, *cAPX*-R: 5’- TGTCCAAGGTCCCTCAAAA -3’; *sAPX*-F: 5’- GTCTGGAGCACATACACTTGGA -3’, *sAPX*-R: 5’- TTAACCGTCCAACGTGAATCCC -3’; Actin-F: 5’-GACCTTGCTGGGCGTGAT-3’, Actin-R: 5’-GTCATAGTCCAGGGCGATGT-3’.
