# Supplementary material for: Proteomic identification of OsCYP2, a rice cyclophilin that confers salt tolerance in rice (Oryza sativa L.) seedlings when overexpressed
Source: BMC Plant Biol. 2011 Feb 16;11:34. doi: 10.1186/1471-2229-11-34 (PMC3050798; doi:10.1186/1471-2229-11-34)
Supplement: Additional file 8 — Correlations between activities of antioxidant enzymes and expression of corresponding genes. [file 1471-2229-11-34-S8.DOC]

**Table S3. Correlations** between activities of antioxidant enzymes and expression of corresponding genes

| Correlation coefficient | SOD | CAT | APX |
| --- | --- | --- | --- |
| *Cu/Zn-SOD* | -0.66 | ND^a^ | ND |
| *Mn-SOD* | -0.42 | ND | ND |
| *Fe-SOD* | -0.23 | ND | ND |
| *OsCat* | ND | 0.41 | ND |
| *OsCatC* | ND | -0.56 | ND |
| *cAPX* | ND | ND | -0.41 |
| *mAPX* | ND | ND | -0.09 |
| *sAPX* | ND | ND | -0.03 |

^a^ND refers to not determined.
